# SNPs in Multi-Species Conserved Sequences (MCS) as useful markers in association studies: a practical approach

**DOI:** 10.1186/1471-2164-8-266

**Published:** 2007-08-06

**Authors:** Jacob L McCauley, Shannon J Kenealy, Elliott H Margulies, Nathalie Schnetz-Boutaud, Simon G Gregory, Stephen L Hauser, Jorge R Oksenberg, Margaret A Pericak-Vance, Jonathan L Haines, Douglas P Mortlock

**Affiliations:** 1 Center for Human Genetics Research and Department of Molecular Physiology and Biophysics, Vanderbilt University Medical Center, Nashville, TN, USA; 2 Genome Technology Branch, National Human Genome Research Institute, National Institutes of Health, Bethesda, MD, USA; 3 Center for Human Genetics and Department of Medicine, Duke University Medical Center, Durham, NC, USA; 4 Department of Neurology, University of California San Francisco, San Francisco, CA, USA; 5 Institute of Human Genomics, Miami University's Miller School of Medicine, Miami, FL, USA

## Abstract

**Background:**

Although genes play a key role in many complex diseases, the specific genes involved in most complex diseases remain largely unidentified. Their discovery will hinge on the identification of key sequence variants that are conclusively associated with disease. While much attention has been focused on variants in protein-coding DNA, variants in noncoding regions may also play many important roles in complex disease by altering gene regulation. Since the vast majority of noncoding genomic sequence is of unknown function, this increases the challenge of identifying "functional" variants that cause disease. However, evolutionary conservation can be used as a guide to indicate regions of noncoding or coding DNA that are likely to have biological function, and thus may be more likely to harbor SNP variants with functional consequences. To help bias marker selection in favor of such variants, we devised a process that prioritizes annotated SNPs for genotyping studies based on their location within Multi-species Conserved Sequences (MCSs) and used this process to select SNPs in a region of linkage to a complex disease. This allowed us to evaluate the utility of the chosen SNPs for further association studies. Previously, a region of chromosome 1q43 was linked to Multiple Sclerosis (MS) in a genome-wide screen. We chose annotated SNPs in the region based on location within MCSs (termed MCS-SNPs). We then obtained genotypes for 478 MCS-SNPs in 989 individuals from MS families.

**Results:**

Analysis of our MCS-SNP genotypes from the 1q43 region and comparison to HapMap data confirmed that annotated SNPs in MCS regions are frequently polymorphic and show subtle signatures of selective pressure, consistent with previous reports of genome-wide variation in conserved regions. We also present an online tool that allows MCS data to be directly exported to the UCSC genome browser so that MCS-SNPs can be easily identified within genomic regions of interest.

**Conclusion:**

Our results showed that MCS can easily be used to prioritize markers for follow-up and candidate gene association studies. We believe that this novel approach demonstrates a paradigm for expediting the search for genes contributing to complex diseases.

## Background

Adding to the challenge of disease gene discovery is the scale of genotyping that is required to conduct association studies in regions demonstrating linkage. Because the follow-up analysis of genomic linkage screen results often entails performing candidate gene analyses within a selected genomic region of interest, the number of necessary genotyping assays to thoroughly test candidate genes within each region quickly becomes very large and often cost prohibitive. New approaches to increase effectiveness of genotyping studies are clearly needed. With this in mind, we provide a novel approach that incorporates evidence from positional and informatic approaches to expedite follow-up of candidate regions. The utility of combining these approaches is evident in several recent studies for complex genetic disorders. For example, the emerging concept of "genomic convergence" suggests that parallel investigations of genetic linkage, association, and expression data will speed disease gene discovery [1]. Application of this process to identify and prioritize candidate genes on chromosome 10 in Alzheimer disease and Parkinson's disease recently led to the successful identification of two genes significantly associated with these diseases [2].

An area of current interest in genomics and disease gene discovery concerns information that can be gained from assessing evolutionary sequence conservation between species. Such sequences that have remained similar across the millions of years of evolution are believed to indicate regions of biological function [3]. Researchers are increasingly integrating knowledge of conserved sequences in the selection of SNPs for genetic studies–whether it is to identify evidence of coding regions, potential splice sites, or regulatory regions [4,5]. In this capacity, conservation can be used as a putative annotation for genomic regions that may have functional importance, even when the precise nature of their function is lacking. This conserved sequence approach has several potential advantages. For example, since noncoding regulatory elements that control neighboring genes can be dispersed across large areas devoid of coding sequences, conserved elements might help discriminate functional regions within large noncoding areas that might not share linkage disequilibrium with coding markers [6-8]. Conservation can also indicate coding regions that may lack strong annotation support, such as alternatively spliced exons, RNA genes, "novel" genes with no homology to other gene families, or genes expressed at very low levels.

A current challenge to genetic association studies is that it is usually not practical to genotype all SNPs in a region of interest. Linkage disequilibrium (LD) patterns can be exploited to identify subsets of "tag" SNPs that can serve as surrogates for detecting associations driven by functional SNPs that are in LD with tag SNPs; however, the statistical power of this approach depends on the degree of LD between markers, and decreases as LD decreases. Testing "functional" SNPs directly should help maximize the likelihood of detecting significant associations with disease phenotypes. By using conservation to prioritize SNPs, the odds may be increased that SNPs impacting the phenotype in question will actually be genotyped. For example, variation in transcriptional levels for key genes can play a significant role in disease risk [5,9]. SNPs in noncoding cis-regulatory sequences, such as enhancers, repressors, or chromatin structural regulators, might contribute to the genetic component of this process by modulating transcriptional output. It is reasonable to predict that SNPs within conserved regions may be more likely to have phenotypic effects than SNPs in nonconserved DNA.

There are currently several publicly available tools to detect evolutionarily conserved sequences across large genomic regions by performing sequence alignments [10,11]. However, simple pair-wise sequence comparisons have drawbacks for use as a systematic approach in prioritization of conserved regions. With a relatively large region, sequence alignment between any two mammalian species can provide too much aligning sequence, resulting in the over-identification of sequences that are not actually preserved due to selective processes and are thus less likely to be functional [12]. Conversely, sequence alignment between more divergent species (e.g. between human and *Fugu*) can provide too little aligning sequence, resulting in the identification of only highly conserved protein-coding regions, while most noncoding regions are unalignable. Fortunately, new alignment methods that compare sequences from multiple species have been developed to minimize these drawbacks. By comparing sequence from three or more vertebrate species, human sequences that are likely to be functional can be detected with improved sensitivity and specificity [13].

Recently, an algorithm for detecting multi-species conserved sequences (MCS) has been optimized for scoring multi-species alignment data across large genomic regions [14]. This allows MCS scores to be assigned across any human genomic region, provided that sequences from multiple species have been aligned. While multiple species are used for comparison, a sequence block can be assigned a high MCS score even if not all the compared species show alignment to the region. This makes the MCS method robust when comparing draft genomes, and for identifying elements that may be conserved only within subgroups of species (e.g. within mammals only). In other words, an MCS may be classified as such even if it is not conserved across all the species in the comparison. MCS analysis incorporates genome sequence alignment data from multiple species in order to assign MCS scores to 50-base pair windows across the human genome. Using this tool, one can identify all sequences in a region of interest that have MCS scores above a defined threshold (e.g., in the top 5% of genome-wide MCS scores) [14]. Substantial portions of multi-species conserved sequences are in noncoding regions. Previous analyses suggest that approximately the top 4–7% of MCS scores are very likely to indicate regions of human DNA undergoing evolutionary selection [14]. This threshold also detects the vast majority of coding exons, yet still detects many noncoding regions. This highlights the importance of MCS in detecting potentially functional sequences within relatively large genomic regions (e.g. several Mb) without relying solely on gene annotation.

We have formulated a systematic approach to prioritize SNP markers to be genotyped for association studies in any target region, with a specific application to the 1q43 linkage region in MS [15,16]. This approach incorporates MCS analysis to prioritize markers within MCSs, termed MCS-SNPs, for high-throughput genotyping. Here we show that annotated SNPs in MCSs were readily identified and are frequently polymorphic. By comparing our findings with publicly available data from the HapMap Project [17] we confirm that MCSs have a slightly reduced SNP density. We also provide an online tool and instructions to extract MCS-SNPs for any region of human DNA, via the UCSC genome browser. Our data is consistent with previous descriptions of human SNP variation in conserved regions [18], suggesting the MCS tool is useful for identifying these SNPs. These findings indicate that conservation is a useful guide for selecting SNPs that may reside in biologically functional elements, particularly for those in noncoding regions.

## Results

We analyzed SNPs in a ~ 7.0-Mb region of human 1q43 that showed suggestive linkage to multiple sclerosis (MS) [15,16]. This region was bounded by SNPs rs10925296 and rs1319790 (encompassing chr1:233,515,650-240,494,277 of human Build 35). We initially defined MCS in this region based on a "4-way" species comparison (see Methods). The majority (478/768) of SNPs that we subsequently genotyped were chosen from within MCS elements (see Methods). An additional 290 SNPs in nonconserved regions were added to provide additional coverage across the 1q43 region. In all, 479 SNPs were within boundaries of known genes and the remaining 289 markers were in intergenic regions. All SNPs were genotyped in our sample population of 173 families (989 individuals). Analyses to test for potential association(s) between these SNPs and multiple sclerosis will be presented elsewhere.

In order to screen as many MCS-SNPs as possible, we selected MCS-SNPs for genotyping without regard to prior validation status. We therefore examined the fraction of our chosen MCS-SNPs and non-MCS-SNPs that demonstrated polymorphism in our study population (Table 1). Approximately 35–37% of SNPs within MCS regions were monomorphic in our study population. Although the percentage of monomorphic SNPs located within non-MCS regions was much less (8–15%), this was likely an artifact of our bias in selection of non-MCS-SNPs. Non-MCS-SNPs were chosen only if there was good prior bioinformatics evidence supporting their validation; however, prior validation was not a criteria used for selecting the MCS-SNPs.

**Table 1:** Descriptive breakdown of SNPs chosen for follow-up genotyping within the 7.0 Mb 1q43 region

Our Dataset	4-way comparison (%)	5-way comparison (%)	8-way comparison (%)
**Total SNPs**	**768 (100%)**	**768 (100%)**	**768 (100%)**
**MCS-SNPs**	**478 (62%)**	**365 (48%)**	**337 (44%)**
Polymorphic	311 (40%)	233 (30%)	213 (28%)
Monomorphic	167 (22%)	132 (17%)	124 (16%)
**non-MCS-SNPs**	**290 (38%)**	**403 (52%)**	**431 (56%)**
Polymorphic	268 (35%)	346 (45%)	366 (48%)
Monomorphic	22 (3%)	57 (7%)	65 (8%)

During our initial study, more genomic sequence data from additional vertebrate species became available. Adding species can potentially increase the sensitivity and specificity of MCS analysis in detecting functional elements [14]. Therefore, we retrospectively compared the fraction of the MCS-SNPs defined by the "4-way" species comparison that were still within MCS as defined by "5-way" and "8-way" species comparisons (see Methods, Table 1). Approximately 70% of MCS-SNPs identified by "4-way" comparison (N = 478) were still within MCSs defined by the "8-way" comparison (N = 337).

We examined the entire ~ 7.0-Mb region in greater detail. Using data from the UCSC genome browser [19] (Build 35) we found over 28,200 distinct annotated SNPs in this region. Of all bases in the 7.0-Mb region, 5.10% were assigned MCS scores that meet the threshold for classification as MCS based on the "8-way" comparison, indicating an MCS density similar to the genome-wide average (defined as the top 5%). However, only ~ 3.2% of the annotated SNPs are within MCS elements. Thus, annotated SNPs are less dense in MCS versus non-MCS sequence. This is consistent with potential selection against variation within regions of high conservation, as observed in a previous whole-genome analysis [18].

These findings indicated that despite the modest reduction in SNP density within MCS, many polymorphic MCS-SNPs were validated in our sample population. Given the recent finding that in general, SNP minor allele frequencies (MAFs) are reduced in conserved sequence [18], we examined HapMap data within the 7.0-Mb interval to determine whether our MCS-SNPs exhibited reduced MAFs [20]. Rates of observed monomorphism were examined, as this could reflect either an excess of very rare alleles in MCS, or that truly polymorphic SNPs were more rare in MCS. Data from the HapMap CEU population (30 trios of northern and western European ancestry collected by the Centre d'Etude du Polymorphisme Humain (CEPH)) was used for comparison, as our sample contained almost exclusively U.S. Caucasian individuals. We counted the total number of HapMap CEU SNPs (N = 11,076 SNPs) falling in MCS regions defined by "8-way" comparisons (N = 28,243 SNPs), and then further subdivided these based on location in or outside exons and whether they were polymorphic or monomorphic in the HapMap CEU population (Table 2). Within the exons of this region, MCS-SNPs had an average MAF of 0.20 and 51% monomorphism rate, while non-MCS-SNPs in exons had an average MAF of 0.27 and 19% monomorphism rate. Thus, as expected exonic MCS-SNPs had reduced MAF and increased monomorphism. Since variation is known to be reduced in coding sequence, we examined the non-exonic SNPs. Similar trends were observed, though less pronounced than in exonic SNPs. Non-exonic MCS-SNPs had an average MAF of 0.22 and monomorphism rate of 37%, while non-exonic/non-MCS SNPs had an average MAF of 0.23 and a 32% monomorphism rate. The difference in MAFs was not significant by chi-square test (not shown), although the trend reflects genome-wide observation [18]. However, the increased rate of monomorphism of MCS-SNPs in non-exonic regions was significant (Table 3).

**Table 2:** Descriptive breakdown of HapMap CEU SNP data across the 1q43 7.0 Mb region

	All annotated SNPs (Build 35) Chr1: 233515650-240494277	CEU population: genotyped SNPs (8-way comparison)
**Total SNPs**	28243	11076*
**exonic SNPs**	208	74
MCS-SNPs	130	47
Polymorphic	NA	23 (MAF = 0.20)
Monomorphic	NA	24
non-MCS-SNPs	78	27
Polymorphic	NA	22 (MAF = 0.27)
Monomorphic	NA	5
**non-exonic SNPs**	28035	11002
MCS-SNPs	776	414
Polymorphic	NA	259 (MAF = 0.22)
Monomorphic	NA	155
non-MCS-SNPs	27259	10587
Polymorphic	NA	7241 (MAF = 0.23)
Monomorphic	NA	3346

**Table 3:** Comparison of monomorphic vs. polymorphic non-exonic SNPs, in HapMap CEU (8-way MCS vs. non-MCS)

		Polymorphic SNPs	Monomorphic SNPs	Chi-square	p-value
**Non-exonic**	MCS	259	155	6.25	0.012
	non-MCS	7241	3346		
**Exonic**	MCS	23	24	7.62	0.006
	non-MCS	22	5		

**Figure 1:**
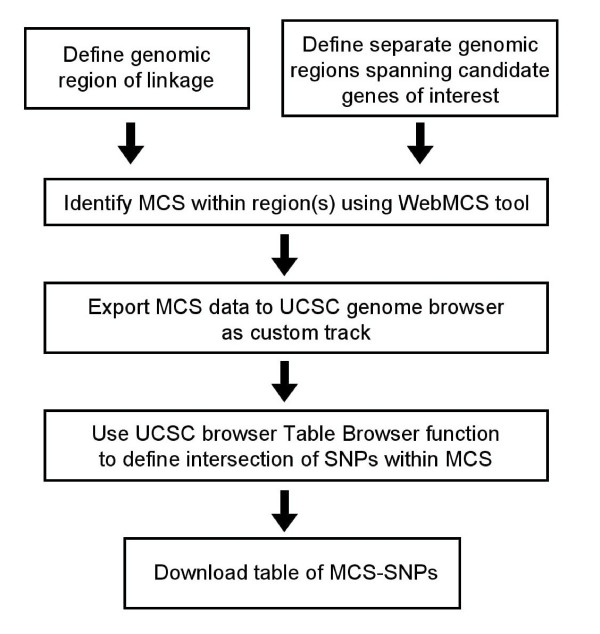
Scheme for identifying MCS-SNPs.

**Figure 2:**
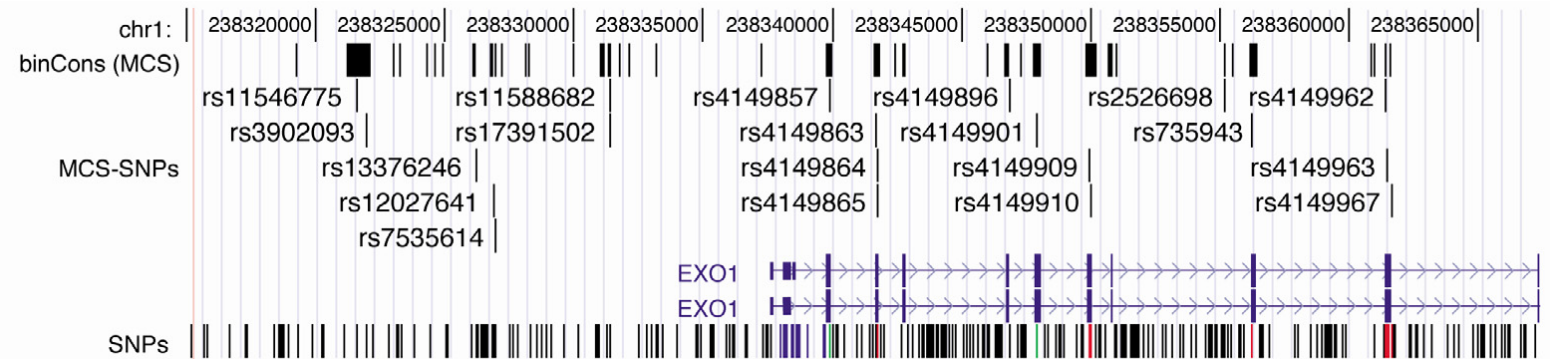
UCSC genome browser image showing position of MCS-SNPs in the vicinity of the *EXO1 *gene. After obtaining the subset of SNPs within MCS, the Table Browser function allows MCS-SNPs to be downloaded in table format or visualized as a custom track on the browser as shown here. Note several MCS-SNPs are in *EXO1 *5' flanking region, and in introns (e.g. rs4149896, rs2526698) as well as those in exons.

An MCS calculator is now available to provide easier access to genome-wide MCS data [21]. This resource allows MCS elements for a human genomic region of interest to be rapidly exported to the UCSC genome browser as a "custom" track, such that SNPs within MCS can be quickly identified (Figure 1). Lists of MCS-SNPs can then be easily retrieved, or visualized on the browser (Figure 2). Detailed instructions, for navigating the WebMCS and UCSC websites in order to extract MCS-SNPs, are provided [see Additional File 1].

Since noncoding MCS-SNPs could affect gene regulation, it may be of interest to prioritize analysis of SNPs that disrupt putative transcription factor binding sites (TFBS). The UCSC browser "TFBS Conserved" track displays predicted TFBS present in regions of human/mouse/rat conservation [19]. This track could be used as an independent SNP filtering mechanism; alternatively, as many predicted sites overlap with MCS it could easily be used to further prioritize MCS-SNPs. For example, in the 7 Mb chromosome 1 region described here 1359 conserved TFBS sites are predicted, of which 889 (65%) are within 8-way MCS regions. For any region of interest, a file of MCS-SNPs could be extracted as a UCSC-ready custom track using the UCSC Table Browser [see Additional File 1], and immediately used to identify the subset of MCS-SNPs that overlap conserved TFBS. In the chromosome 1 region 30 HapMap SNPs were found to overlap TFBS; of these, 15 are also within 8-way MCS.

Actual coverage of MCS-SNPs in genotyping studies depends on the coverage of working genotyping assays. For the latter reason, the coverage of MCS-SNP assays in existing whole-genome SNP genotyping platforms is of interest. For both chromosomal regions, we determined the fraction of MCS-SNPs that are in existing Affymetrix 500 K SNP chips and in the Illumina 300 K assay set (Table 4). Interestingly, coverage for these assay sets ranges from roughly 4–12% but was roughly twice as great in the chromosome 8 region, independent of platform. Therefore, the large majority of MS-SNPs are not directly represented in these genotyping platforms. For genotyping genomic regions of interest, the effective association coverage of MS-SNPs with these assay sets will be a function of the MS-SNP representation for the assay set used, the ability of assays in the set to "tag" LD across markers, and the LD structure of the population in question.

**Table 4:** Coverage of 8-way MCS-SNPs in whole-genome assay sets

	Chr1: 233,515,650-240,494,277	Chr8: 95,000,001–100,000,000
Total MCS-SNPs	906	382
	within MCS:	
Affymetrix 500 K Nsp SNPs	31 (3.4%)	29 (7.6%)
Affymetrix 500 K Sty SNPs	21 (2.3%)	14 (3.7%)
Total Affy500 K	52 (5.7%)	43 (11.7%)
Illumina 300 K	39 (4.3%)	32 (8.4%)

Since LD can be used to select tag SNPs that provide surrogate information for groups of SNPs having shared LD, it is reasonable to question whether prioritization of MCS SNPs is useful if LD tagging strategies can "cover" MCS regions effectively. To assess this, we used the Tagger tool [22] through the HapMap website [20] to obtain HapMap CEU tag SNPs for the 7 Mb region (using MAF > = 0.05, r2 > = 0.8). This analysis was also performed for a randomly chosen 5 Mb region on chromosome 8 roughly centered on the *GDF6 *gene (build hg17, chr8: 95,000,001–100,000,000). This region contains a higher gene density than the 7 Mb chromosome 1 region (30 RefSeq annotated genes; ~ 170 kb/gene) but slightly lower 8-way MCS coverage (3.73%). For the chromosome 1 and 8 regions, respectively, 242 and 146 SNPs fell in MCS; of these, 34 (14%) and 13 (9%) did not share LD with any other SNPs and were thus not captured by other tags. Therefore, while a large majority of HapMap-genotyped MCS-SNPs are captured by tag SNPs obtained by these parameters from the CEU data set it may be of interest to augment tagging strategies by including "uncaptured" MCS-SNPs in genotyping studies.

## Discussion

Here we show that polymorphic SNPs can be readily identified within conserved noncoding regions, and that SNP selection based on conservation data is an approach that can be readily incorporated into genotyping projects. Genetic association studies have typically placed heavy emphasis on coding SNPs, with the idea that these are more likely to have biological impact than noncoding SNPs. SNP analysis in noncoding DNA has typically been restricted to promoter regions, though conservation in more distant noncoding regions is a widespread observation. Our approach prioritizes SNPs in conserved noncoding and coding regions, since conservation is widely accepted to correlate with function in either type of sequence.

Although the density of MCS in the ~ 7.0-Mb region is similar to the genome-wide average, the number of exonic bases and total genes per kb are below genome averages. The region contains 17 genes based on RefSeq annotation [19], which is far below the genome average of approximately 9 genes per Mb [23]. Notably, this region contains several large genes (*RYR2*, 791 kb; *RGS7*, 582 kb; *PLD5*, 436 kb) as well as a "gene desert" of ~ 1.5-Mb. Based on RefSeq annotation, 51.3% of genomic bases in the interval are spanned by transcription units, and 1.02% of bases are within exons, less than the estimated genome-wide fraction of coding bases (1.5%) [12]. Even with the conservative assumption that 100% of exon bases fall within MCS elements, at least 4% of MCS bases must be in noncoding MCS regions (see Results). Since not all exon bases are in MCS regions, this is a minimal estimate. Thus, relative to the whole genome the 7.0-Mb interval has slightly below average density of genes and exonic bases, but has a fraction of noncoding MCS that is similar to genome-wide estimates of noncoding conservation [12].

There are two similar hypotheses at play when discussing variation in regions of conservation. The first is that regions of conserved DNA sequence, whether coding or noncoding, are likely to have biological function(s). The extension of this idea is that when examining these regions in suspected disease genes, functional variations will be discovered that predispose to disease risk. However, a second hypothesis is that because conserved regions are indeed likely to have important functions, variants that persist within these regions are actually those that are less likely to have functional effects, as variants that impact function are likely to reduce fitness and be subjected to negative selection pressure. This stems from the concept that most perturbations in conserved sequence will reduce reproductive fitness. These conflicting hypotheses can be somewhat reconciled by the notion that many genetic variants that predispose to complex, common diseases in the present day may have had negligible effects on reproductive fitness in recent human evolution. Regions of high conservation harbor biological function and therefore are likely to be under selective constraint. New variants in these regions are often detrimental and thus less likely to persist in populations, which may explain the increased rate of apparent monomorphism (or rare alleles) for MCS markers. However, some persisting MCS variants may have phenotypic effects that influence disease risk. Variants that alter potential TFBS are particularly interesting and may be worthwhile to prioritize, although the density of such MCS-SNPs was on the order of one per gene in the chromosome 1 region. However, since actual binding sites can deviate from the predicted consensus and not all factor binding motifs are known, not all "functional" SNPs that disrupt true binding sites will be identified with this approach.

For certain studies, different combinations of species might be more desirable for classifying MCS. For example, inclusion of additional mammals might be useful for detecting functional elements that are not expected to be present in other vertebrates. In fact, diseases that involve the immune response (such as MS or lupus) might be controlled by genes with a more recent or dynamic evolutionary history. We note that users can define MCS with custom-generated sequence alignments based on species data of their choice, using the WebMCS online resource [24].

Since many MCS-SNPs are not represented in widely used whole-genome genotyping platforms, the inclusion of MCS-SNPs is particularly for candidate gene regions where dense coverage of potentially functional SNPs is of greater interest. For candidate genomic regions (or populations) that are characterized by low linkage disequilibrium, prioritizing MCS-SNPs may have additional value, as markers are less likely to be effectively captured by tagging strategies.

## Conclusion

Our results are consistent with recent reports that SNP density and derived (new) SNP allele frequencies are slightly reduced in noncoding conserved regions as compared to nonconserved regions [18]. Our findings also confirm that inferences from genome-wide conservation data can be usefully applied to SNP selection for fine-mapping genetic studies. The MCS-SNP approach represents practical knowledge for choosing SNP markers and can be integrated with current approaches for genotyping studies to refine or follow-up regions or genes believed to be involved in disease.

## Methods

### Subjects and phenotypes

This study involved genotyping 768 SNPs in a dataset of 173 multiple sclerosis families (989 individuals genotyped). Families were ascertained at the University of California at San Francisco (UCSF) as previously described [15]. Of these 173 families, 91 families had previous evidence for positive linkage to 1q43 [16]. Informed consent was obtained for all subjects. This research was performed under protocol #8692 as approved by Vanderbilt University Internal Review Board.

### Molecular analysis

768 SNPs were chosen for genotyping by selecting SNPs from the UCSC genome browser with significant preference given to markers with an MCS score exceeding the top 5% threshold (see below). The Illumina BeadArray™ platform was used for SNP genotyping. All genotyping was performed by the Duke Genomics Resource Laboratory Core using the Illumina BeadArray™ platform.

For this study we used MCS scores that fall in the top 5% of genome-wide MCS scores for 50-base pair sequences [14], based on "4-way" genome-wide alignment between human, mouse, rat, and chick genomes (see below). Selection of SNPs in conserved regions was based on informativeness (with preference given to SNPs with high minor allele frequencies), validation (preference to SNPs confirmed by multiple lines of evidence), location (preference to SNPs spaced at regular intervals), putative function (preference to SNPs in coding, splice site, and mRNA UTR regions), and assay scores provided by Illumina (preference to SNPs generating scores > 0.60). Illumina scores were determined by an algorithm weighing a series of factors to predict the success of each locus within an OPA. Scores ranged between 0 and 1, with Illumina recommending selection of SNPs generating scores > 0.60.

Several SNPs located in 5% MCS regions were eliminated from the study due to the nature of the variation precluding genotyping on the Illumina platform (e.g. insertion/deletions or multiple mutation events leading to > 2 alleles) and/or the failure to generate an Illumina score > 0.60. Because elimination of these SNPs resulted in the identification of fewer than 768 SNPs in conserved regions, additional SNPs were selected from nonconserved regions, with similar consideration for suitability to the Illumina platform and to create an average spacing of < 10 kb for the 768 SNPs in the ~ 7.0-Mb region of interest. This density was chosen to make most efficient use of the Illumina platform and may help to exploit linkage disequilibrium for future association studies, based on LD patterns in Caucasians [25]. Most markers fell within intronic and intergenic areas.

### Statistical analysis

We initially identified multi-species conserved sequences (MCSs) through a "4-way" alignment of mouse, rat, and chick genomic sequence to human chromosome 1q43 sequence (Build 35, chr1:233,515,650-240,494,277) [14,21]. MCS regions were defined as those regions with MCS scores falling in the top 5% of genome-wide scores. This allowed us to identify 478 SNPs from MCS regions that were amenable to genotyping on the Illumina platform as described above. Since the time we initially chose the markers, additional genome sequences for other model organisms have been produced. Therefore we also analyzed our SNPs using new MCS scores derived from both "5-way" alignments of genomic sequences (human, mouse, rat, chick, and *Fugu*) and "8-way" species alignments (human, chimp, mouse, rat, dog, chick, *Fugu*, and zebrafish) (Margulies, unpublished). Currently, genome-wide MCS data based on the "8-way" species comparison can be obtained online [21].

HapMap CEU data from the international HapMap project, were used to examine SNP data in the 1q43 region (HapMap Data Rel#20/phaseII Jan06, on NCBI B35 assembly, dbSNP b125; [20]). Comparisons of numbers of monomorphic and polymorphic markers in each group were made using a Chi-square test for significance (Table 3). The UCSC browser TFBS conserved track was implemented with a Z score cutoff of 1.64 [19].

## Authors' contributions

JLM analyzed genotype and HapMap data and prepared the draft manuscript. SJK helped conceive the study design, selected SNPs and conducted genotyping. NSB and SGG helped coordinate and direct sample handling and genotyping. EHM created the WebMCS tool. SLH and JRO directed patient ascertainment and oversaw blood sample collection. MPV, JLH, and DPM helped conceive and organize the project and helped draft the manuscript.

## Supplementary Material

Additional file 1 Supplementary instructions on how to determine, view, and extract MCS-SNPs for a region of interest. This file provides a step-by-step tutorial for obtaining MCS-SNPs from a given region of the human genome. Click here for file
