# Supplementary material for: SNPs in Multi-Species Conserved Sequences (MCS) as useful markers in association studies: a practical approach
Source: BMC Genomics. 2007 Aug 6;8:266. doi: 10.1186/1471-2164-8-266 (PMC1959193; doi:10.1186/1471-2164-8-266)

Additional file 1.
Supplementary information:
Instructions for obtaining human SNPs in MCS regions.

Currently, genome-wide MCS data is available for human build 35 (May 2004).

1. Go to URL: **http://zoo.nhgri.nih.gov/binCons/**

2. Under the pull-down menus, select genome build "hg17" (May 2004 / build 35) and track "8way_binMCS_95.bed" .

3. Input UCSC genome browser coordinates, for the region of interest (hg17/May 2004 / build 35).

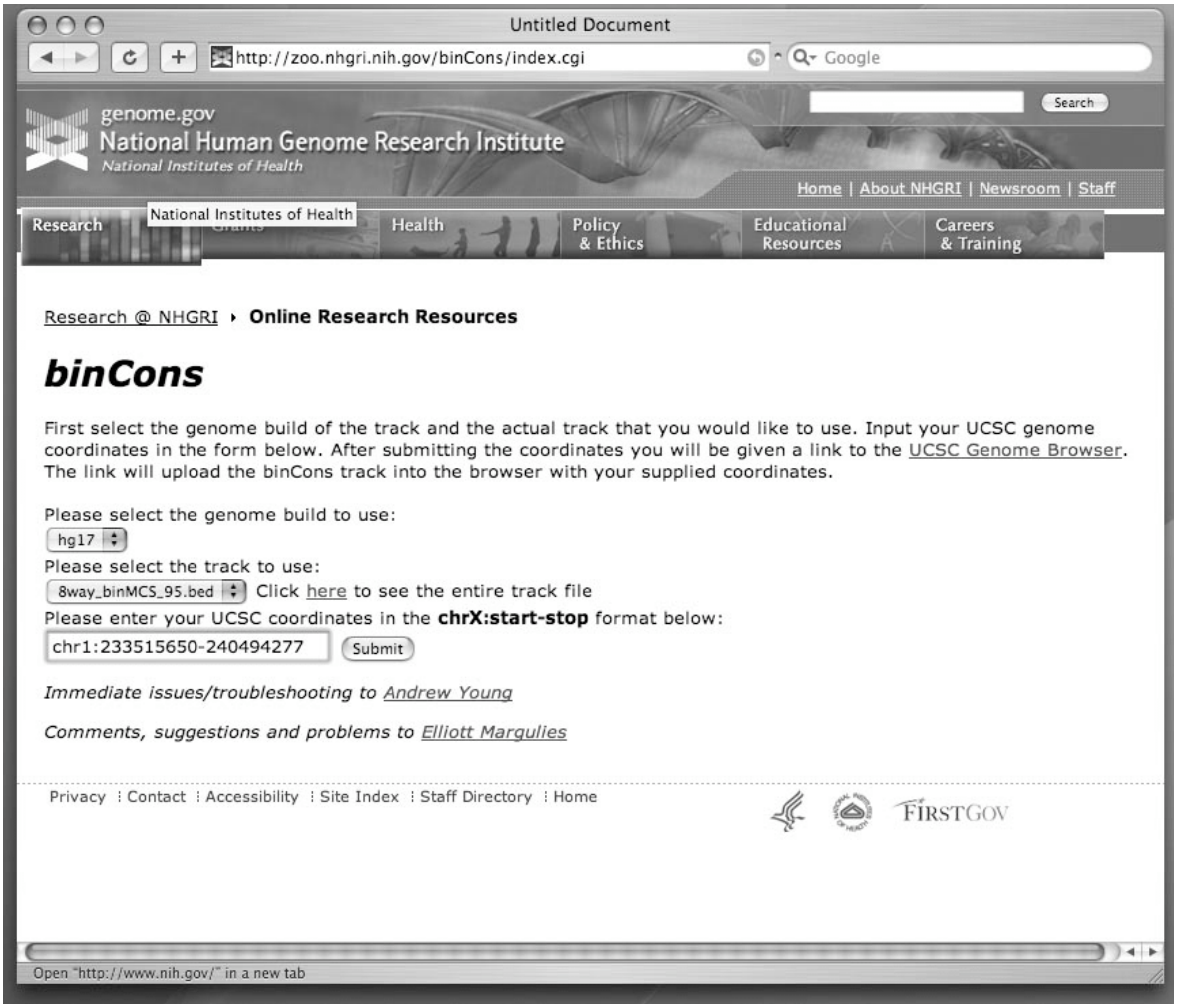

4. After new page loads: under the “Output” header, click the “here” link to be directed to the UCSC genome browser. The region of interest should be displayed. MCS data will automatically be pre-loaded into a custom track on the browser. (The MCSs can be visually inspected, although this is usually not practical for large genomic regions.)

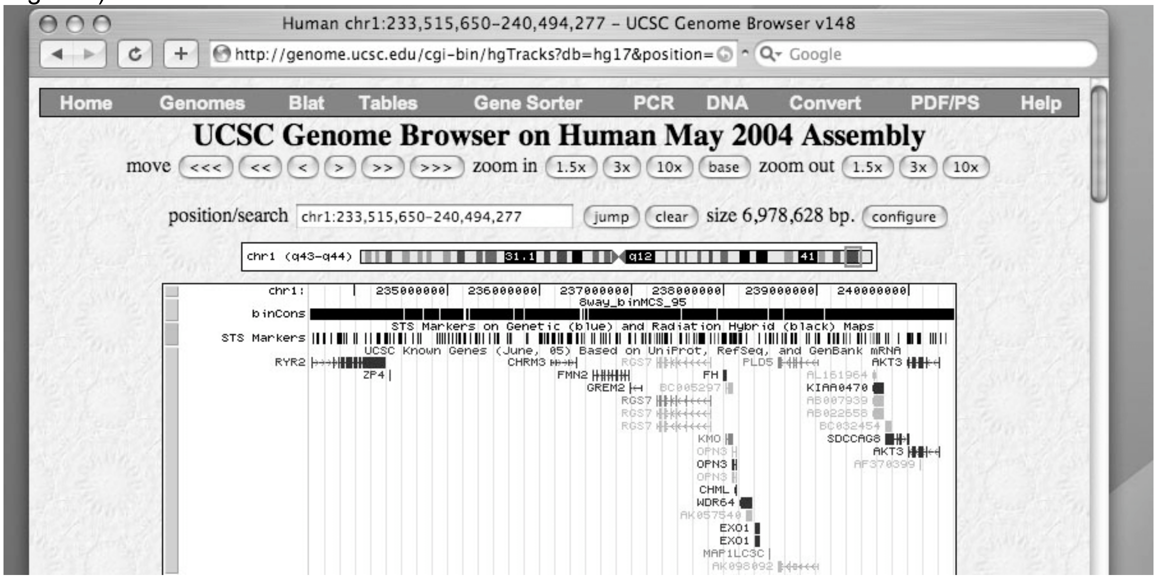

5. To download a table of only those SNPs in the region of interest that are within MCSs, click the “Tables” link at top of the browser page to link to the “Table browser” page.

6. Note, the coordinates for the region of interest should now be listed in the “region” window next to the “position” radio button.
Under “Group” menu, select “Variation and repeats”.
Under “Track” menu, select “SNPs”.
Next to “Intersection”, click the “create” button.

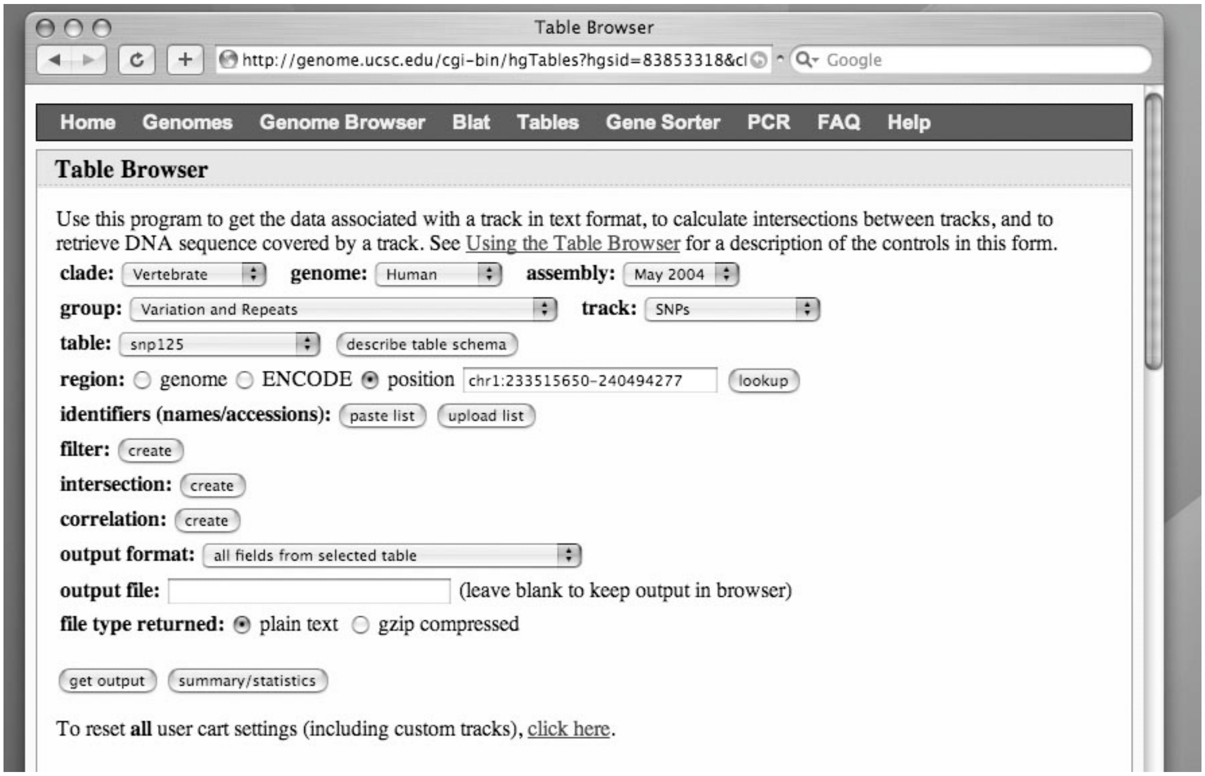

7. On the following page:
Under “group”, select “Custom tracks”.
Under “track”, select “binCons”.
Select the radio button “All SNPs records that have any overlap with binCons”.
Click “submit” to be returned to the Table browser page.

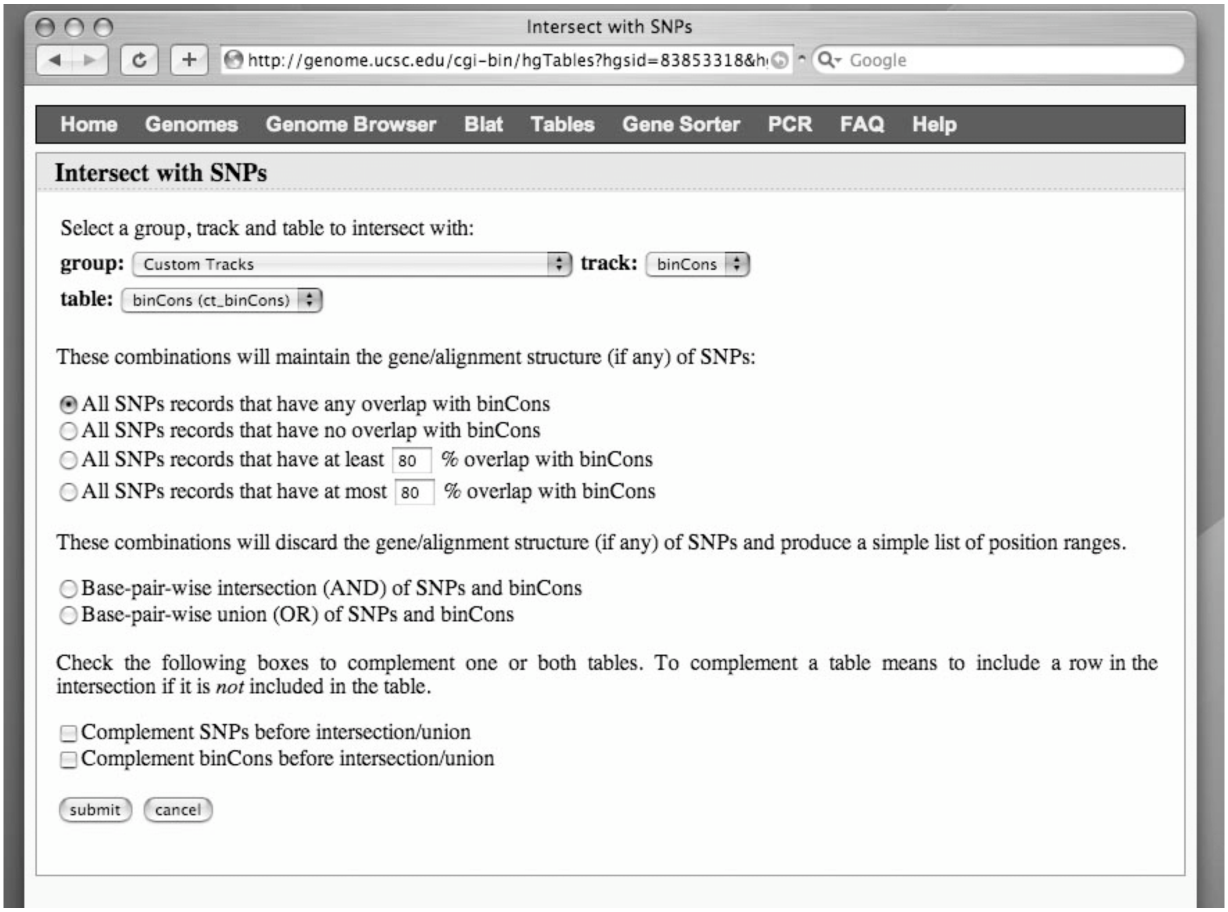

8. Under “output format”, select “BED – browser extensible data”. (Alternatively, select “custom track” to get the data in UCSC custom track format.)

Click “get output”.

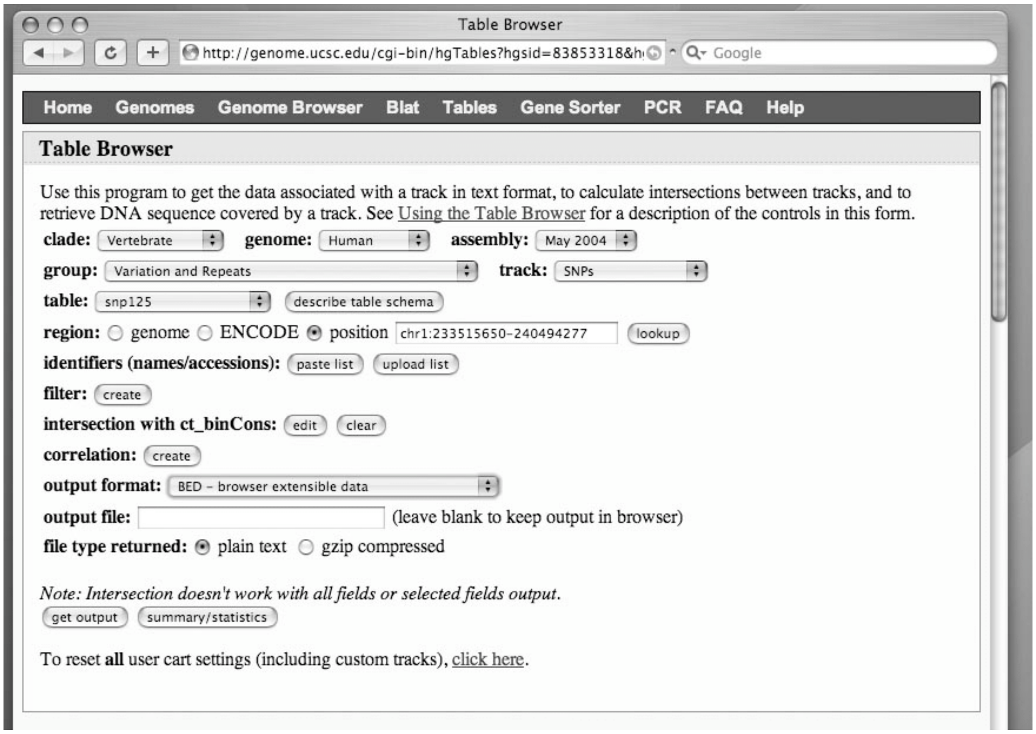

9. On the following page click “get BED”. This returns a table with SNP coordinates and rs numbers.

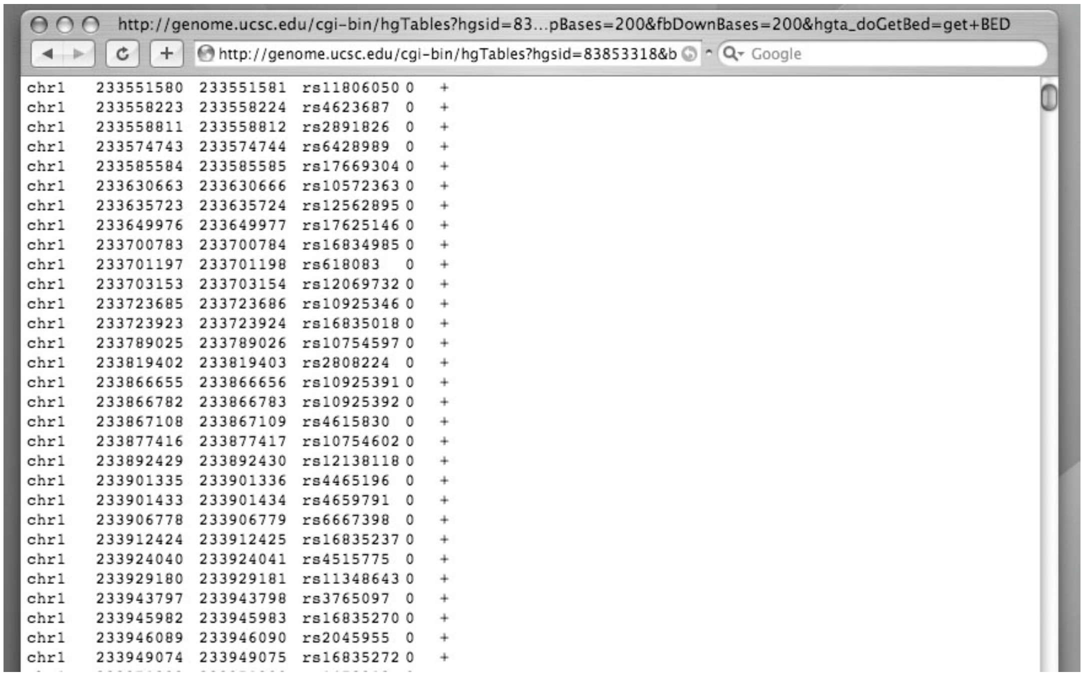

Supplement: Additional file 1 — Supplementary instructions on how to determine, view, and extract MCS-SNPs for a region of interest. This file provides a step-by-step tutorial for obtaining MCS-SNPs from a given region of the human genome. [file 1471-2164-8-266-S1.pdf]
